# Rationale and pre-clinical evidences for the use of autologous cartilage micrografts in cartilage repair

**DOI:** 10.1186/s13018-018-0983-y

**Published:** 2018-11-06

**Authors:** Marco Viganò, Irene Tessaro, Letizia Trovato, Alessandra Colombini, Marco Scala, Alberto Magi, Andrea Toto, Giuseppe Peretti, Laura de Girolamo

**Affiliations:** 1 IRCCS Istituto Ortopedico Galeazzi, via Riccardo Galeazzi 4, 20161 Milan, Italy; 2 Human Brain Wave, corso Galileo Ferraris 63, 10128 Turin, Italy; 3 Primus Gel srl, Via Casaregis, 30, 16129 Genoa, Italy; 4 Clinica Veterinaria San Rossore, via delle cascine 149, 56100 Pisa, Italy; 5 Department of Biomedical Sciences for Health, Università degli Studi di Milano, via Mangiagalli 31, 20133 Milan, Italy

**Keywords:** Micrografts, Cartilage repair, Regenerative medicine, Cartilage defects, Rigenera

## Abstract

**Background:**

The management of cartilage lesions is an open issue in clinical practice, and regenerative medicine represents a promising approach, including the use of autologous micrografts whose efficacy was already tested in different clinical settings. The aim of this study was to characterize in vitro the effect of autologous cartilage micrografts on chondrocyte viability and differentiation and perform an evaluation of their application in racehorses affected by joint diseases.

**Materials and methods:**

Matched human chondrocytes and micrografts were obtained from articular cartilage using Rigenera® procedure. Chondrocytes were cultured in the presence or absence of micrografts and chondrogenic medium to assess cell viability and cell differentiation. For the pre-clinical evaluation, three racehorses affected by joint diseases were treated with a suspension of autologous micrografts and PRP in arthroscopy interventions. Clinical and radiographic follow-ups were performed up to 4 months after the procedure.

**Results:**

Autologous micrografts support the formation of chondrogenic micromasses thanks to their content of matrix and growth factors, such as transforming growth factor β (TGFβ) and insulin-like growth factor 1 (IGF-1). On the other hand, no significant differences were observed on the gene expression of type II collagen, aggrecan, and SOX9. Preliminary data in the treatment of racehorses are suggestive of a potential in vivo use of micrografts to treat cartilage lesions.

**Conclusion:**

The results reported in this study showed the role of articular micrografts in the promoting chondrocyte differentiation suggesting their potential use in the clinical practice to treat articular lesions.

**Electronic supplementary material:**

The online version of this article (10.1186/s13018-018-0983-y) contains supplementary material, which is available to authorized users.

## Background

The management of cartilage lesions still represents a challenge for surgeons, due to the limited regenerative ability of cartilage, given its avascularity and hypocellularity. Additionally, cartilage defects can lead to the pathogenesis of osteoarthritis, resulting in pain and disability with a high economic and social impact in many developed countries [1]. Depending on the type of cartilage defect, different methodologies for cartilage repair and regeneration can be applied today, such as arthroscopic debridement [2], bone marrow stimulations [3], osteochondral autografts, or allograft [4]. Beyond these methodologies, autologous chondrocyte implantation (ACI) or matrix-induced autologous chondrocyte implantation (MACI) can represent valid approaches in the treatment of cartilage defects, to promote the formation of hyaline or hyaline-like cartilage and improve the pain and functional outcomes in most of treated patients. However, nowadays, the use of autologous chondrocytes is limited by the need to perform two-stage procedures and by the long recovery time required after surgery [5].

Recently, regenerative medicine approaches, based on cell therapy or tissue engineering, gather increasing interest, representing possible therapeutic alternatives. Indeed, the use of live cells with appropriate scaffold and growth factors could allow for restoration of physiological tissue within small or large defects. Nevertheless, recent studies demonstrated that progenitors cells from cartilage are the preferred cell type for this kind of approach, since other cell types, such as bone marrow-derived mesenchymal stem cells, are not able to generate hyaline cartilage [6].

The Rigenera® procedure is an innovative clinical protocol to obtain autologous articular micrografts, containing both live cells and fragments of hyaline cartilage matrix, ready to use alone or in combination with the most common scaffolds. Previous studies have been reported that autologous micrografts are enriched of progenitor cells and maintain regenerative properties [7, 8]. The autologous micrografts are already used in dentistry [9, 10]and wound healing [11–15], reporting satisfactory results in terms of bone and dermal regeneration. Furthermore, some recent papers showed the effectiveness of micrografts also in the cartilage and cardiac regeneration [16, 17].

In this study, we provide an in vitro characterization of cartilage autologous micrografts properties on chondrocytes viability and differentiation, together with an evaluation of their pre-clinical application in racehorses affected by joint diseases, where autologous micrografts were used in combination with autologous platelet-rich plasma (PRP) to better vehicle the micrografts in the injured site and obtain a biocomplex ready to be used.

Joint diseases represent the main cause of reduced athletic function for racehorses and are characterized by a degenerative process involving several components of the joints including cartilage, subchondral bone, and articular capsule [18]. Anti-inflammatory and analgesic drugs represent the standard treatment for mild defects, while articular cartilage curettage, osteophyte removal or surgical arthroscopy, and arthrodesis can be indicated for severe cartilage and bone degeneration [19]. Nevertheless, while all these therapies effectively reduce symptoms, they are not able to restore the physiological conditions in cartilage tissue. Regenerative therapy for racehorses is assuming a growing interest for the significant economic impact on the horse industry, and racehorses can be a valuable large animal model for the evaluation of new therapies due to the interspecies similarities with humans in the thickness of the non-calcified cartilage of the stifle joint [20].

## Materials and methods

### Isolation of human primary chondrocytes and culture with micrografts

Human primary chondrocytes were isolated from eight samples of articular cartilage of femoral head of donor patients undergoing total hip arthroplasty. All individuals provided informed consent as per the Institutional Review Board approved procedure (M-SPER-014.ver7). Primary chondrocytes were isolated from articular cartilage (0.6–1.2 mg) using overnight incubation at 37 °C with 0.15% *w*/*v* type II collagenase (Worthington, NJ, USA) solution in DMEM (Sigma Aldrich, MO, USA) + 5% fetal bovine serum (FBS, Hyclone, Thermo-Fisher Scientific, MA, USA). Cells were then seeded at 5.000 cell/cm^2^ for expansion. The autologous micrografts were obtained by Rigenera protocol after mechanical disaggregation using a medical disposable Rigeneracons (Human Brain Wave srl, Turin, Italy) [9]. Briefly, 200 mg of each sample was inserted in the Rigeneracons and minced for 5 min in a total of 5 ml of DMEM. The primary chondrocytes isolated by collagenase were cultured in four different conditions: DMEM supplemented with 10% FBS (control medium), control medium plus 10% *v*/*v* autologous micrografts, DMEM supplemented with 1% FBS and chondrogenic factors (chondrogenic medium), and chondrogenic medium plus 10% *v*/*v* autologous micrografts. For cell viability assay, only control medium and control medium with 10% *v*/*v* autologous micrografts were tested. Particles obtained after disaggregation with Rigenera ranged from 50 to 70 μm.

### Cell viability

Cell viability was assessed at 1, 4, 7, and 14 days of incubation with the different media by MTT [3-(4,5-dimethylthiazol-2-yl)-2,5-diphenyltetrazolium bromide, Sigma-Aldrich] assay. Cells at passage 3 were cultured in 96-well plates at the density of 3.0 × 10^3^ cells/cm^2^; to perform the assay, a final concentration of 0.5 mg/mL MTT was added to the culture medium and incubated for 4 h at 37 °C; the medium was removed and 100% DMSO was added to each well to solubilize the precipitate. Absorbance was read at 570 nm.

### Chondrogenetic differentiation assay

For chondrogenic differentiation, 5.0 × 10^5^ cells were centrifuged at 250*g* for 5 min to obtain pellets. The pellets were cultured in four different media: control medium, DMEM supplemented with 100 U/ml penicillin, 100 μg/ml streptomycin, 0.29 mg/ml L-glutamine, 1 mM sodium pyruvate, 1.25 mg/ml human serum albumin (HAS; Sigma-Aldrich), and 10% FBS; chondrogenic medium, consisting of DMEM supplemented with 100 U/ml penicillin, 100 μg/ml streptomycin, 0.29 mg/ml L-glutamine, 1 mM sodium pyruvate, 1.25 mg/ml human serum albumin (HAS; Sigma-Aldrich), 1% ITS+1 containing 1.0 mg/ml insulin from bovine pancreas, 0.55 mg/ml human transferrin, 0.5 μg/ml sodium selenite, 50 mg/ml bovine serum albumin and 470 μg/ml linoleic acid (Sigma-Aldrich), 0.1 μM dexamethasone, 0.1 mM L-ascorbic acid-2-phosphate, and 10 ng/ml TGF-β1 (PeproTech, Rocky Hill, NJ, USA) (Lopa S); control medium plus 10% *v*/*v* autologous micrografts; chondrogenic medium plus 10% *v*/*v* autologous micrografts. The medium was replaced every 3 days and cells cultured at 37 °C under a 5% CO_2_ atmosphere for 4 weeks before the following evaluations.

### Histology and immunohistochemistry

For the histological analysis, representative pellets from each sample and treatment (*n* = 32) were fixed in 10% neutral buffered formalin (Bio-Optica Milano SpA, Milan, Italy), embedded in paraffin blocks, and cut into 4-μm-thick sections. To detect sulfated glycosaminoglycans (GAGs), sections were stained with standard Alcian blue protocol (Bio-Optica). Briefly, slides were deparaffinized and rehydrated then stained with Alcian blue (pH 2.5; according to Mowry) for 30 min. The sections were then immersed in a sodium tetraborate solution for 10 min and counterstained with Mayer’s hematoxylin, dehydrated, and mounted.

For immunohistochemical localization of collagen type I (COLL I) and collagen type II (COLL II), the sections were dewaxed and rehydrated, and a heat-induced antigen retrieval was applied using a microwave treatment for 5 min at 400 W in citrate buffer pH 6.0 (Thermo Fisher Scientific, Waltham, MA, USA). Then, the slides were treated with 3% H_2_O_2_ in absolute methanol for 10 min to quench endogenous peroxidases and successively with 3% *w*/*v* bovine serum albumin (BSA) in PBS for 30 min to inhibit non-specific reactivity. Biotinylated anti-COLL I (10 μg/ml; #7026, Chondrex Inc., Redmond, WA, USA) and biotinylated anti-COLL II (10 μg/ml; #7049, Chondrex Inc.) antibodies were applied overnight at 4 °C in a humid chamber upon sections. The primary antibodies were diluted in PBS with 1% *w*/*v* BSA and 0.3% *v*/*v* Tween 20 (Thermo Fisher Scientific). At the end of incubation, biotinylated antibodies were detected with streptavidin conjugated to horseradish peroxidase (Abcam, Cambridge, UK) and then with HIGHDEF® yellow IHC chromogen (Enzo Life Sciences Inc., Farmingdale, NY, USA). All sections were finally weakly counterstained with Mayer’s hematoxylin, dehydrated, and mounted. For negative control, the primary antibody was omitted. Photomicrographs were taken with an Olympus IX71 light microscope and an Olympus XC10 camera (Japan).

### GAGs deposition

Glycosaminoglycans (GAG) content was evaluated by dimethylmethylene blue (DMMB) assay. Briefly, the pellets were digested at 60 °C for 16 h in PBE buffer (100 mM Na2HPO4, 10 mM Na EDTA, pH 6.8) containing 1.75 mg/ml L-cysteine (Sigma-Aldrich) and 14.2 U/ml papain (Worthington, Lakewood, NJ, USA). The obtained extracts were incubated with 16 mg/l dimethylmethylene blue (Sigma-Aldrich), and absorbance was read at 500 nm (Perkin Elmer Victor X3 microplate reader). For normalization purposes, DNA content evaluation was performed on each sample by CyQUANT Kit (Life Technologies), following manufacturer’s instructions. Data are presented as microgram of GAGs per microgram of DNA.

### Quantitative real time-PCR

Total RNA was isolated from cells using TriReagent (Life Technologies) and cDNA was synthesized from 1 μg of total RNA by reverse transcription (RT) reaction. The expression of type II collagen (*COL2A1*), *SOX9*, and aggrecan (*ACAN*) mRNAs was measured by real-time RT-PCR, using TaqMan reagents (Life Technologies). The calculations of the results were carried out according to the 2^ΔCt^ methods. GAPDH was used as an internal control for data normalization [21]**.**

### Growth factors measurement

Immediately after micrograft production with the Rigenera protocol, an aliquot of the micrograft suspension was frozen at − 20 °C. Transforming growth factor β (TGFβ) and insulin-like growth factor 1 (IGF-1) concentrations in the micrograft suspensions were measured by commercially available ELISA kit, according to the manufacturer’s instructions (Peprotech, UK).

### Horses

Three horses (1 gelding and 2 thoroughbreds) aged from 3 to 5 years (4.4 ± 1.5) with intra-articular lesions were treated. Their characteristics, before and after treatment, are provided in Table 1.

**Table 1:** Baseline characteristics of horses before and after treatment

Horses	Sex and breed	Diagnosis	AAEP lameness scale (before treatment)	AAEP lameness scale (after treatment)
1	Gelding, quarter horse	Middle-carpal joint arthrosis, severe cartilage erosions, and detachment on radial and third carpal bone, radial bone fragments	3	0
2	Male, thoroughbred	Severe cartilage damage to the metacarpal-phalangeal joint, joint space reduction, early signs of bone proliferation associated with degenerative osteoarthritis at early stage	3	0
3	Female, thoroughbred	Cartilage ulcer of the dorsomedial eminence of the first phalanx. Linear erosions of metacarpal condyles. Cartilage thinning	2	1

*AAEP* American Association of Equine Practitioners

### Preparation of equine platelet-rich plasma (PRP)

Autologous PRP was prepared as previously described [22]. Briefly, two units of 450 ml of blood are collected from the horses through a standard triple-bag system, a method that allows easily the removal of 450–900 ml of blood. Sampling was done from the jugular vein after trichotomy and disinfection of the area. Blood was centrifuged at 1450 rpm for 10 min at 20 °C, in order to obtain the separation of red blood cells from plasma containing platelets and the factors that lead to the formation of a clot. Plasma is then centrifuged at 3000 rpm for 20 min at 20 °C, thus obtaining the separation of a platelet pellet and platelet-poor plasma (PPP). The platelets are then re-suspended in 30–35 ml of PPP in order to have a PRP with a platelet concentration of about 1 × 106 platelets/μl. The bag containing the PRP is placed on a platelet agitator under constant agitation at room temperature and after about 2 h transferred under a sterile hood to dispense the platelet concentrate into sterile tubes (Monovette, Sarstedt). The PRP product is stored at − 20 °C until use.

### Use of equine autologous micrografts and PRP in arthroscopy interventions

**Fig. 1:**
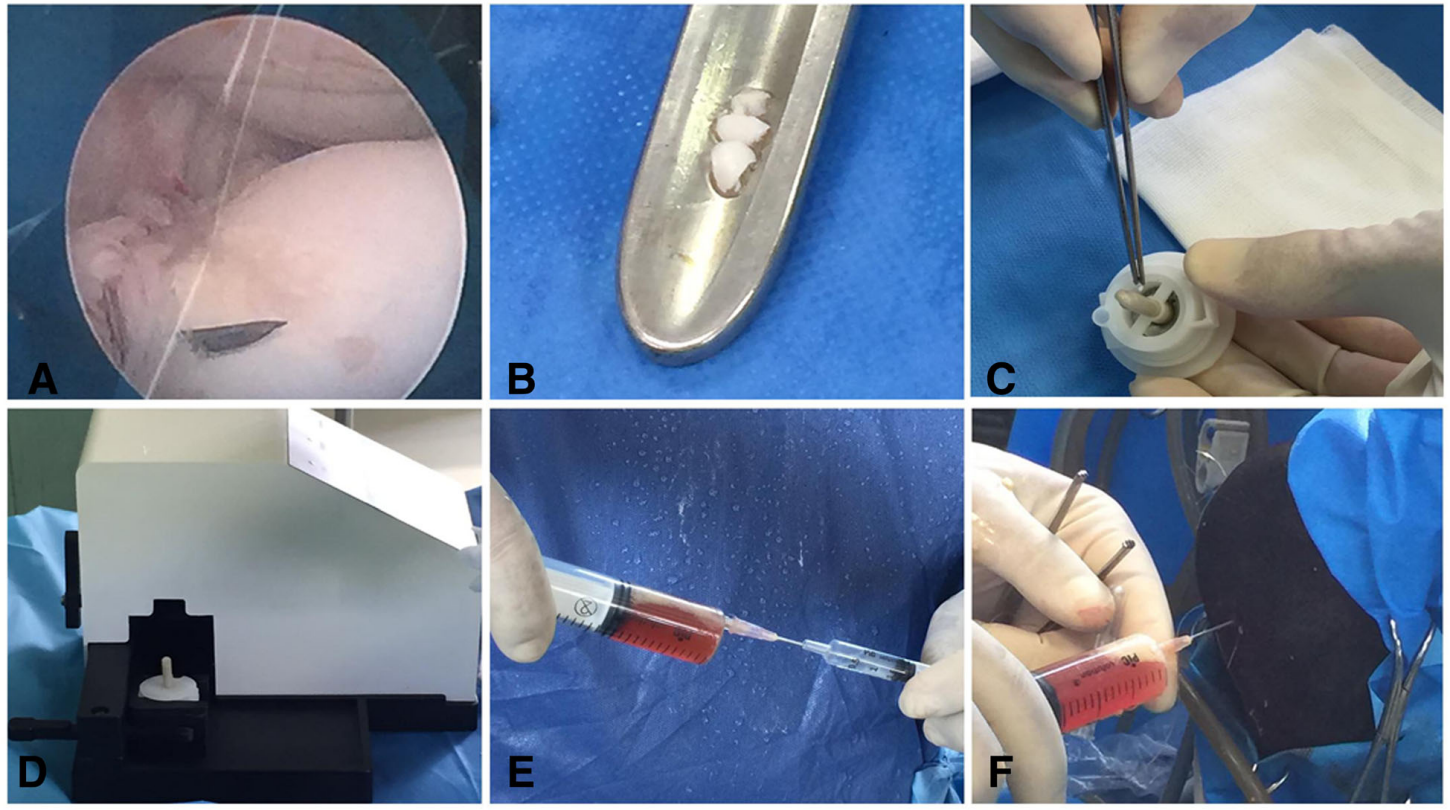
Collection of equine autologous micrografts. **a**, **b** During the arthroscopy intervention, a small piece of intra-articular cartilage was collected by endoscopic procedure. **c**, **d** The intra-articular cartilage was inserted in Rigeneracons disposable and disaggregated for 2 min by a rotation process triggered by Rigenera machine. **e**, **f** The micrografts obtained were mixed to autologous PRP and directly injected on the lesion

Equine autologous micrografts were prepared as previously described using Rigenera protocol. Briefly, a small piece of intra-articular cartilage (weight 0.0230 g) was collected by endoscopic procedure and disaggregated by Rigeneracons medical device for 5 min for three times adding 1.5 ml of sterile physiological solution (Fig. 1a–d). The chondrocyte-derived micrografts were then mixed to 10 ml of PRP (Fig. 1d) and after an arthroscopic curettage injected in the articular lesions of horses (Fig. 1e). After the procedure, the skin is disinfected with iodine product and dressed with cotton gauze and a Vetrap-type bandage strip. The dressing remains in situ for 48 h. A clinical follow-up was performed every week, while the radiographic follow-up was performed between 4 and 6 months after arthroscopy.

### Statistical analysis

All data are reported as means ± SD. Statistical difference between two groups was determined by one-way ANOVA or *t* test, when appropriate. The significance was established for a *p* value ≤ 0.05.

## Results

### Effect of autologous micrografts on chondrocyte differentiation and viability

**Fig. 2:**
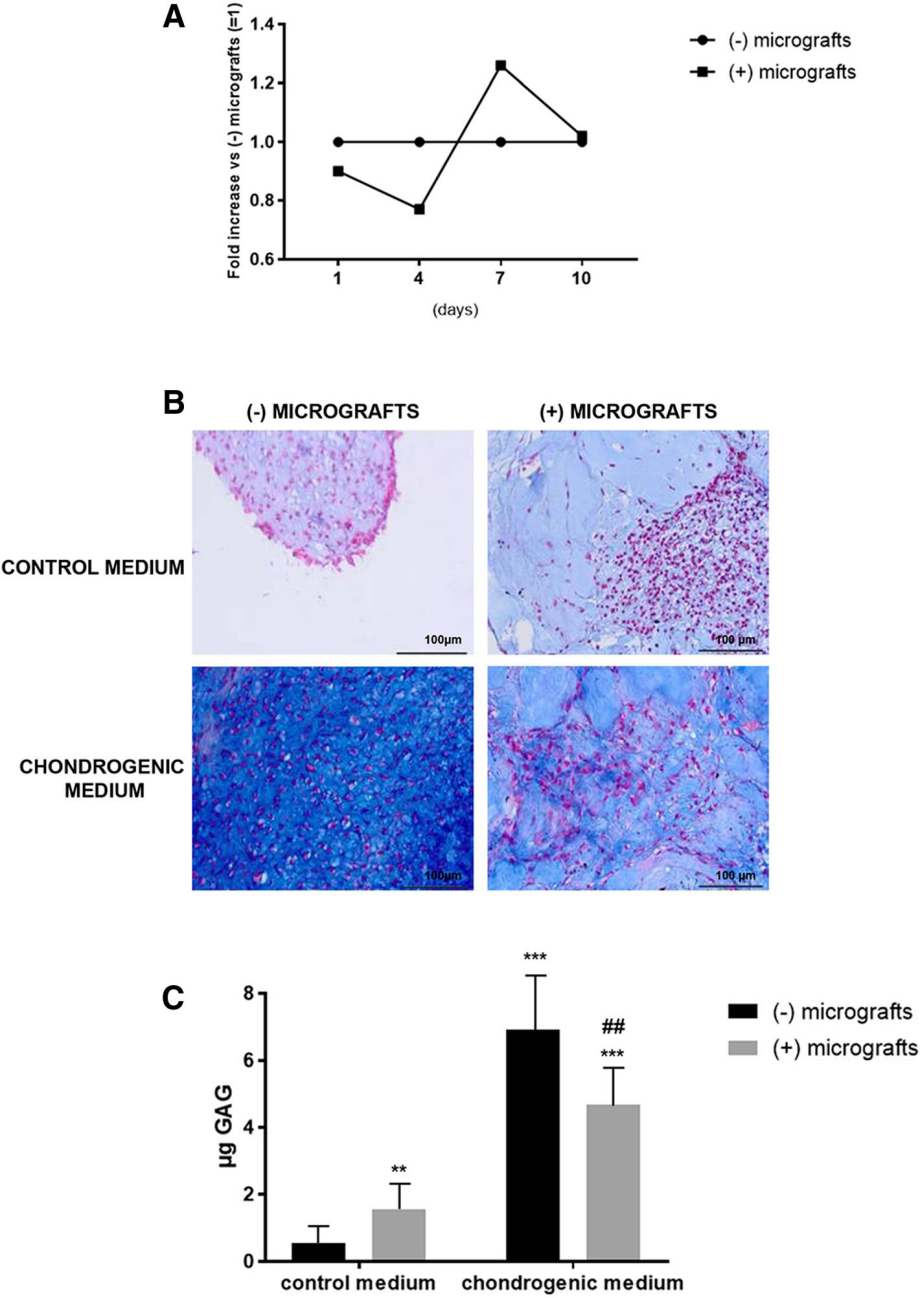
Chondrogenic differentiation. **a** Cell viability evaluated after 10 days of culture in the presence or absence of autologous micrografts. The results are expressed as fold increase with respect to chondrocytes cultured without micrografts (= 1). **b** Alcian blue staining to evaluate the chondrogenic differentiation of cells cultured in the control or chondrogenic medium with or without autologous micrografts (magnification × 20). **c** GAGs deposition in cells cultured in the control or chondrogenic medium with or without autologous micrografts (**p < 0.01, ***p < 0.001 vs cells in the control medium without micrografts; ## *p* < 0.01 vs cells in the chondrogenic medium without micrografts)

The effect of micrografts on cell viability was evaluated after 10 days of culture, and no significant interference was observed comparing cells cultured in the presence and absence of cartilage micrografts (Fig. 2a). The chondrocyte differentiation was evaluated by histology and immunohistochemistry on human primary chondrocyte pellets cultured in the presence or absence of autologous micrografts. The micromasses cultured in the presence of micrografts showed higher dimension and positivity to Alcian blue staining with respect to control samples. When cultured in a chondrogenic medium, these differences were less obvious, but the action of micrografts in favoring cell harboring was confirmed (Fig. 2b). In addition, a DMMB assay revealed that GAGs content was significantly increased in the cells cultured with micrografts in standard culture conditions (*p* < 0.01) (Fig. 2c). Nevertheless, a decrease was observed in chondrogenic medium plus micrografts cultured pellets with respect to samples maintained in chondrogenic medium alone. Despite this difference resulted statistically significant (*p* < 0.01), the differentiation ability was not prevented as demonstrated by the great increase in GAGs content observed in these pellets when compared to control cells (*p* < 0.001) (Fig. 2c).

**Fig. 3:**
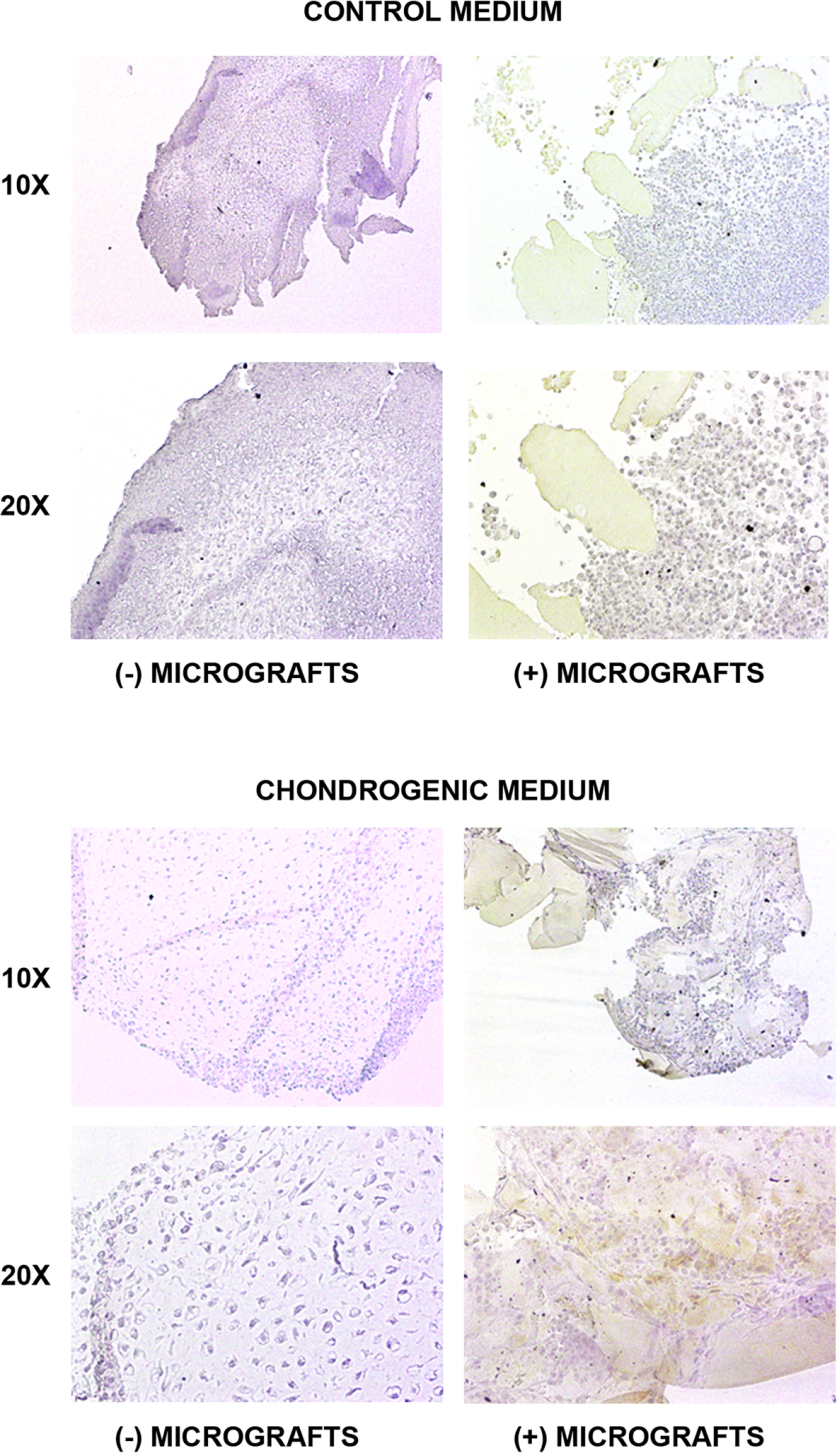
Type II collagen expression. The type II collagen expression was evaluated by immunohistochemistry in the cells cultured both in the control or chondrogenic medium in the presence or absence of autologous micrografts after 4 weeks of culture (magnification × 10 and × 20)

**Fig. 4:**
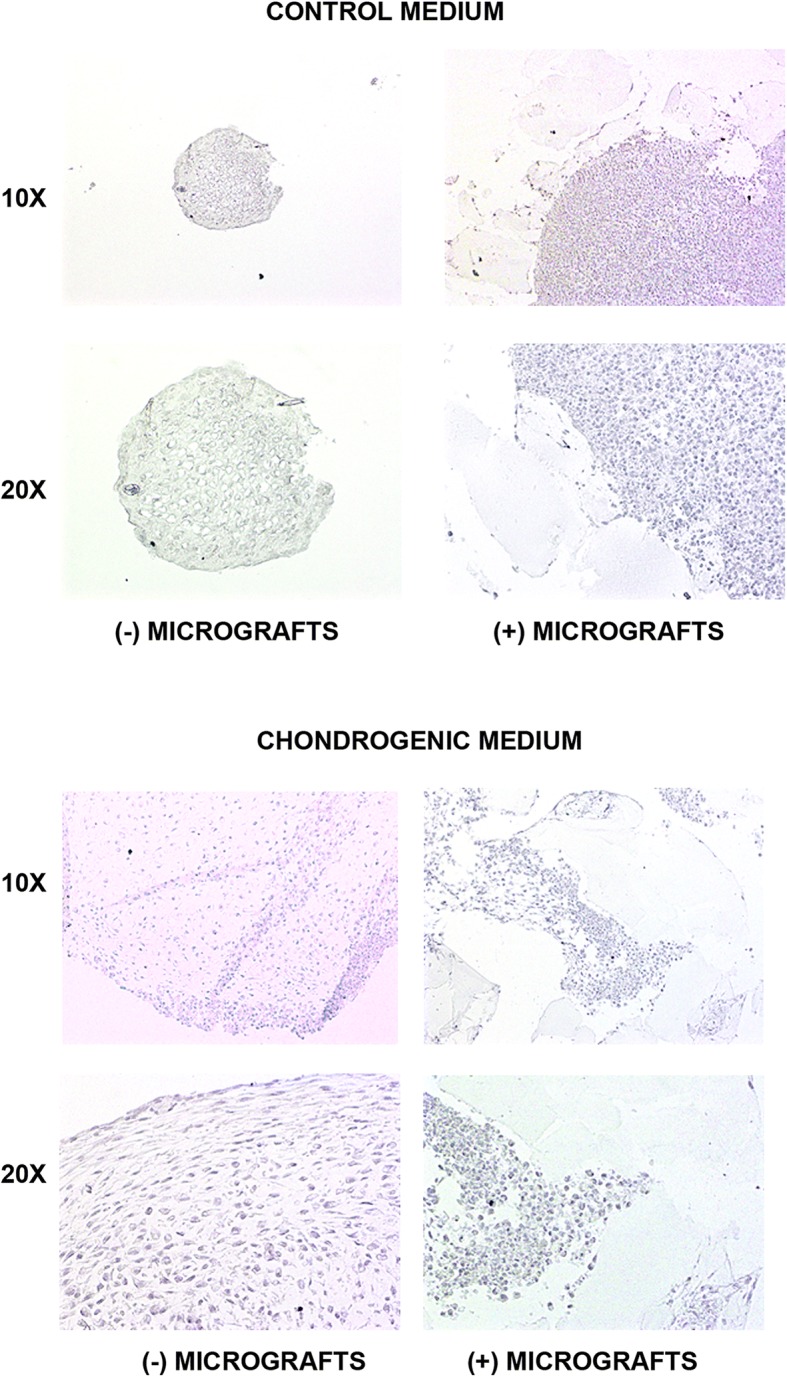
Type I collagen expression. The type I collagen expression was evaluated by immunohistochemistry in the cells cultured both in the control or chondrogenic medium in the presence or absence of autologous micrografts after 4 weeks of culture (magnification × 10 and × 20)

Finally, immunohistochemistry analysis revealed a strong presence of type II collagen in the chondrocytes plus micrografts with respect to those without micrografts, both in the control or chondrogenic medium (Fig. 3). As expected, the cells are negative for type I collagen (Fig. 4), confirming the lack of trans-differentiation events.

### Evaluation of the cartilage-specific gene expression

**Fig. 5:**
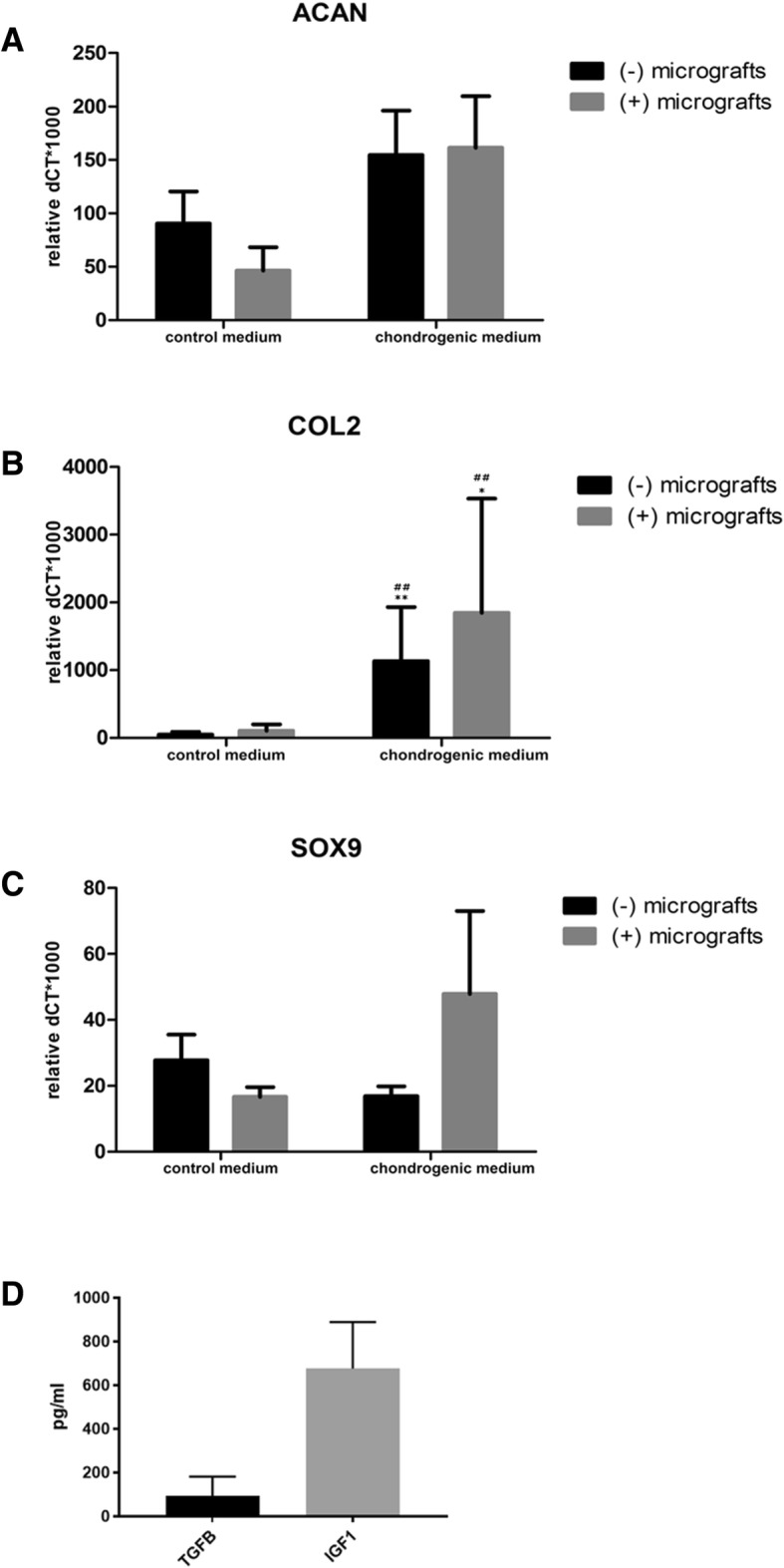
Gene expression of ACAN, COL2, and SOX9 and measurement of cartilage trophic factors TGFβ and IGF-1. **a**–**c** mRNA expression of ACAN (aggrecan), COL2 (type II collagen), and SOX9 was evaluated in the cells cultured both in the control or chondrogenic medium in the presence or absence of autologous micrografts after 4 weeks of culture. The results are expressed as dCt vs. GAPDH. **d** The levels of cartilage trophic factors TGFβ and IGF-1 were measured by ELISA in the autologous micrografts suspension. *p < 0.05, **p < 0.01 vs control medium (−) micrografts; ^##^*p* < 0.01 vs control medium (+) micrografts

Type II collagen and aggrecan are critical components for cartilage structure. We evaluated their expression in human primary chondrocytes showing that in the chondrogenic medium, the expression of these markers increases with respect to samples cultured in control medium, in the presence or absence of autologous micrografts. The expression of the cartilage-specific transcription factor SOX9 resulted more expressed in samples treated with chondrogenic medium and micrografts, with respect to all the other culture conditions. Nevertheless, due to the high inter-donor variability, no statistically significant differences were found in the gene expression of these markers (Fig. 5a–c).

### Autologous micrografts suspension contains cartilage trophic factors

The suspension of the autologous micrografts was assayed for cartilage trophic factors TGFβ and IGF-1. The mean content of TGFβ in all micrografts samples was 81.4 ± 88.2 pg/ml, while IGF-1 was 676.3 ± 212.7 pg/ml. All samples showed the presence of IGF-1, while only six out of eight samples showed the presence of TGFβ (Fig. 5d).

### Use of autologous micrografts plus PRP for arthroscopic intervention in the sport racehorses

**Fig. 6:**
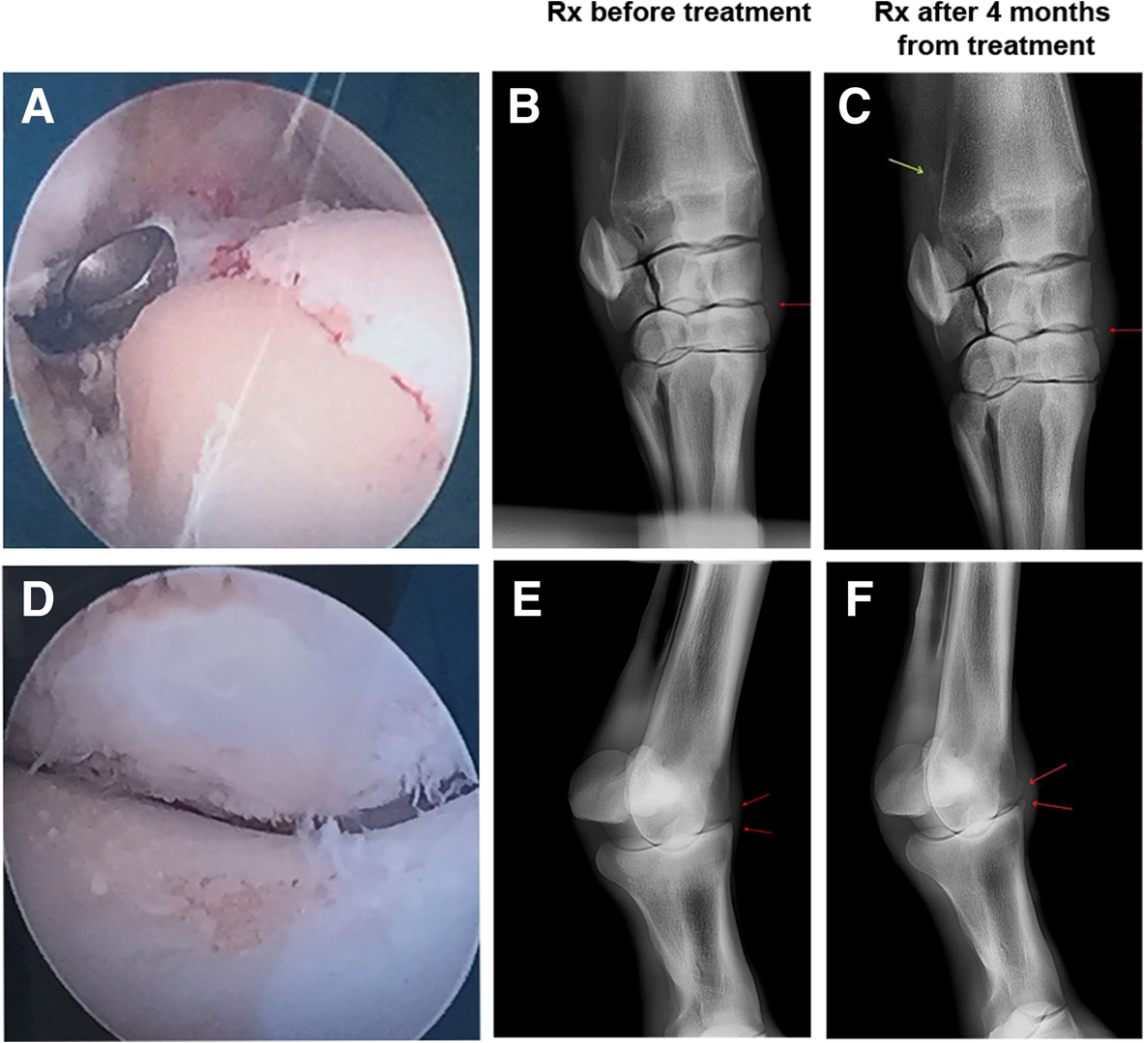
Application of equine autologous micrografts in arthroscopy intervention. **a** Endoscopic image of left intercarpal joint showing the surface of the radial bone of the carpus after curettage and removal of the large portion of damaged cartilage. **b**, **c** Right front carpus, dorsolateral palmaro-medial X-ray pre- and post-intervention which evidences the radio-carpal joint, the middle carpal joint, and the proximal extremities of the metacarpal bones. Osteophyte on the dorsomedial edge of radial bone (yellow arrow) and articular fragment close to the third carpal bone (red arrows). **d** Endoscopic image of right metacarpal-phalangeal joint showing a severe damage to the articular cartilage. At the bottom, at the level of the lateral condyle of third metacarpal bone, and at the top, on the lateral portion of the proximal side eminence of the phalanx. **e**, **f** Right fetlock, dorsolateral palmaro-medial X-ray pre- and post-intervention which reports the metacarpal-phalangeal joint. Articular space and signs of bone proliferation before and after treatment (red arrows)


Autologous micrografts and PRP were used for arthroscopic intervention in sports racehorses affected by joint disease causing lameness. In the first horse, a severe cartilage erosion was observed (Fig. 6a) and the RX pre-intervention showed the presence of both middle-carpal arthrosis and an osteophyte on the dorsomedial edge of the radial bone with an articular fragment close to the third carpal bone (Fig. 6b). After 4 months from intervention, an improvement of articular, dorsomedial, and inter-carpal edge can be observed (Fig. 6c). In the second horse, a severe cartilaginous damage was observed (Fig. 6d) and the RX pre-intervention showed a reduced articular space and an early stage of degenerative arthrosis (Fig. 6e). After 4 months from micrografts plus PRP injection, an increase of articular and peri-articular proliferation was observed (Fig. 6f). For the third horse, the diagnosis of cartilagenous damage on the fetlock was confirmed by clinical evidences but not at a radiographic level showing no difference before and after the treatment (data not shown). In two cases, a complete resolution of lameness which allowed the recovery of sports race activity was observed (Additional files 1 and 2).

Additional file 2: Resolution of lameness after micrograft application. Videos showing the activity of horses 1 and 3 (referring to Table 1) after 4 months from micrografts application. (MP4 10901 kb)


## Discussion

The possibility to use biological strategies to enhance the cartilage regeneration ability in a one-step surgery would represent an important advance in the treatment of cartilage defects. Indeed, while the most used techniques are nowadays limited to microfractures for bone marrow stimulation or two-step surgeries for articular chondrocyte transplantation, the use of Rigenera® procedure would improve the feasibility of biological treatments in the field. The in vitro results reported in this study demonstrate that autologous micrografts do not affect chondrocyte viability and influence chondrocyte differentiation, as shown by both increased GAGs deposition and the presence of collagen II in primary human cells cultured in the presence of micrografts supporting the formation of chondrogenic micromasses and acting like a scaffold for chondrocyte harboring. From our experiments, the presence of IGF-1 and TGFβ in the product obtained by cartilage processing with Rigenera protocol also emerged; both factors were able to enhance cartilage repair in vivo by increasing proteoglycan synthesis and stimulating mesenchymal stem cell differentiation into chondrocytes, stimulate matrix synthesis, and reverse the catabolic effects of pro-inflammatory cytokines [23, 24].

Previous in vitro studies reported that micrografts maintained a high cell viability after mechanical disaggregation of different types of human tissues such as dental pulp, periosteum, and cardiac atrial appendage [7] and that they are able to differentiate in osteocytes, adipocytes, and chondrocytes [8].

In addition to in vitro data, the study provides positive preliminary results in the treatment of racehorses affected by joint diseases, suggesting an in vivo application of cartilage micrografts associated with PRP. The efficacy of micrografts in the cartilage repair was reported in previous human studies where the authors described the combined the use of autologous chondrocyte-derived micrografts and PRP to reconstruct not hyaline alar nasal cartilage and to promote cartilage regeneration in patients affected by external nasal valve collapse. In fact, the constructs of chondrocyte micrografts-PRP resulted in a persistent cartilage tissue with appropriate morphology, adequate central nutritional perfusion without central necrosis or ossification, and further augmented nasal dorsum without obvious contraction and deformation [16, 25].

To confirm the clinical efficacy of micrografts in the tissue repair/regeneration, several case series studies were performed in different clinical areas such as dentistry, dermatology, and wound care. To this regard, it has been reported that human dental pulp or periosteum-derived micrografts were able to promote the bone regeneration in the atrophic maxilla [9], to preserve the alveolar socket after tooth extraction by both reducing bone resorption and increasing new bone formation [10] and to promote sinus lift augmentation [26]. Autologous micrografts also improve the wound healing of complex post-operative and post-traumatic wounds [27] of post-surgical dehiscences [11, 12] and chronic ulcers [13, 14]. Furthermore, micrografts were also used in the treatment of pathological and hypertrophic scars restoring the structural layers immediately below the epidermis and promoting the horizontal realignment of collagen fibers in the papillary dermis [15]. Finally, a very recent paper showed the ability of autologous micrografts to induce cardiac regeneration [17].

Joint healing can be improved by supplying stem cells, growth factors, and growth substrates at the site of injury. For example, several studies demonstrate that mesenchymal stem cells were effective in the bone and cartilage regeneration due their capacity to differentiate into osteocytes and chondrocytes and stimulate the synthesis of the chondrocyte extracellular matrix [28]. In previous studies, we showed that cells contained in the micrografts derived from different tissues express mesenchymal stem cells (MSCs) markers, such as CD90, CD73 and CD105, CD117, and CD44 [7, 8, 25], suggesting the presence of MSCs or tissue-specific progenitors within micrografts, as a possible explanation of their regenerative potential. Behind the stem cells, also the PRP containing both PDGF and TGF-β1 was shown to be effective in cartilage regeneration by promoting chondrocyte proliferation and synthesis of proteoglycan and type II collagen [29]. We reported in this study the pre-clinical application of autologous micrografts combined with PRP in the cartilage repair in racehorses, suggesting that a combined action of both these factors would be able to promote the osteochondral regeneration. In fact, PRP alone resulted effectively in the treatment of cartilage lesions only at short-term [30, 31], and it has been suggested that the combination with other approaches would allow for the stabilization of its beneficial effects [29]. Even if the known short-term effect of PRP may have masked the benefits of autologous cartilage micrografts, our in vivo data provide the first proof of concept for the use of the combination of these techniques in cartilage defects. Moreover, the use of osteochondral autografts and allograft is already a well-established practice in the management of horse injuries, allowing the treatment of large defects, thanks to the immediate reconstruction of the articular surface by transfer of mature intact hyaline cartilage and the underlying subchondral bone. The success of this technique depends on the viability of chondrocytes in the graft and on the mechanical stability of the host–graft interface [32]. Furthermore, the use of osteochondral grafts transfer is limited by donor site availability in the autologous approach or joint congruency and host response in the case of allogeneic tissue [19]. The use of autologous micrografts overcomes some of these limitations, given that their collection is scarcely invasive, reducing donor site morbidity without influencing the grafts viability and overcoming the possible rejection issue related to allogeneic grafts.

## Conclusion

Taken together, these results showed that autologous cartilage micrografts may promote cartilage repair, favoring chondrocytes harboring and growth, and they suggest their potential in the treatment of articular lesions in combination with PRP. However, further studies are needed to confirm the effectiveness of micrografts on cartilage repair/regeneration both alone or in combination with different approaches.

## Additional files

Additional file 1: Resolution of lameness after micrograft application. Videos showing the activity of horses 1 and 3 (referring to Table 1) after 4 months from micrografts application. (MP4 2050 kb)

## Abbreviations

*ACAN*: Aggrecan
ACI: Autologous chondrocyte implantation
BSA: Bovine serum albumin
*COL2A1*: Type II collagen
COLL I: Collagen type I
COLL II: Collagen type II
DMMB: Dimethylmethylene blue
DMSO: Dimethyl sulfoxide
FBS: Fetal bovine serum
GAGs: Glycosaminoglycans
HAS: Human serum albumin
IGF-1: Insulin-like growth factor 1
MACI: Matrix-induced autologous chondrocyte implantation
MTT: 3-(4,5-Dimethylthiazol-2-yl)-2,5-diphenyltetrazolium bromide
PPP: Platelet-poor plasma
PRP: Platelet-rich plasma
TGFβ: Transforming growth factor β
